# Differences in assistive technology installed for people with dementia living at home who have wandering and safety risks

**DOI:** 10.1186/s12877-021-02546-7

**Published:** 2021-10-30

**Authors:** Eleanor Curnow, Robert Rush, Sylwia Gorska, Kirsty Forsyth

**Affiliations:** School of Health Sciences, Queen Margaret University, Edinburgh, EH21 6UU UK

**Keywords:** Living situation, Caregiver support, Secondary data analysis, Living alone

## Abstract

**Background:**

Assistive Technology for people with dementia living at home is not meeting their care needs. Reasons for this may be due to limited understanding of variation in multiple characteristics of people with dementia including their safety and wandering risks, and how these affect their assistive technology requirements. This study therefore aimed to explore the possibility of grouping people with dementia according to data describing multiple person characteristics. Then to investigate the relationships between these groupings and installed Assistive Technology interventions.

**Methods:**

Partitioning Around Medoids cluster analysis was used to determine participant groupings based upon secondary data which described the person characteristics of 451 people with dementia with Assistive Technology needs. Relationships between installed Assistive Technology and participant groupings were then examined.

**Results:**

Two robust clustering solutions were identified within the person characteristics data. Relationships between the clustering solutions and installed Assistive Technology data indicate the utility of this method for exploring the impact of multiple characteristics on Assistive technology installations. Living situation and caregiver support influence installation of assistive technology more strongly than level of risk or cognitive impairment. People with dementia living alone received different AT from those living with others.

**Conclusions:**

Results suggest that caregiver support and the living situation of the person with dementia influence the type and frequency of installed Assistive Technology. Reasons for this include the needs of the caregiver themselves, the caregiver view of the participants’ needs, caregiver response to alerts, and the caregiver contribution to the assistive technology assessment and selection process. Selection processes should be refined to account for the needs and views of both caregivers and people with dementia. This will require additional assessor training, and the development of validated assessments for people with dementia who have additional impairments. Policies should support the development of services which provide a wider range of AT to facilitate interventions which are focused on the needs of the person with dementia.

## Background

Safety and wandering risks are associated with adverse outcomes for people with dementia [1], and are identified as particular areas of concern for people with dementia and their caregivers [2, 3]. Wandering has been identified as the third biggest cause of accidental injury for people with dementia [1]. Falls-related fractures [4], anxiety or caregiver impact, nursing home admission, alongside the resources used whilst searching for missing persons with dementia are viewed as major adverse outcomes associated with wandering [5, 6].

Assistive technology (AT) has been proposed as an intervention which can reduce the risk of adverse outcomes related to safety and wandering, by meeting the needs of people with dementia. However, there is an acknowledged gap between required care and the AT services provided for people with dementia [7], and evidence for their effectiveness remains inconsistent [8]. The reasons for this are unclear but perhaps include incomplete awareness of differences in the requirements of people with dementia in the real world [9], insufficient assessment of their circumstances [10, 11], and limited availability of AT interventions [12, 13].

Variations of the model of healthcare utilisation indicate that many characteristics have an impact upon health service use [14–16]. Characteristics associated with the acceptance of AT include positive perceptions of the technology, level of anxiety, perceived benefit, choice, level of cognitive impairment, gender, living situation and social support [17–19]. However, the relative importance of each characteristic, their impact, together with the heterogeneity of user requirements and other person characteristics restricts understanding of their relationship to AT interventions [20–23]. Research into the effects of multiple variables on the provision of AT is scarce [20]. Need factors have traditionally been viewed as the most immediate cause of health service use [24]. However, predisposing characteristics including the relationship between the person with dementia and their caregiver are important predictors of health care utilisation [16]. Additionally, enabling resources such as caregiver support can facilitate or inhibit the use of healthcare services [25].

Unmet needs and risks are strongly associated with adverse outcomes [1, 26], and wandering and safety risks have been identified as primary concerns for caregivers of people with dementia [2, 3]. As there is evidence that these risks can be modified, this study will focus upon AT installed to reduce risks in these areas [1].

In order to provide people with dementia with effective, client centred AT interventions service providers must understand patterns of need for people with dementia and how these relate to specific AT interventions [20, 27, 28]. Hence, there is a need to explore the relationship between multiple variables and AT use.

This research aims to investigate patterns in person characteristics of people with dementia living at home, specifically: wandering and safety risks; Mini Mental State Examination scores (MMSE) [29]; living situation; caregiver support; and how these relate to installed AT.

## Methods

This study used secondary analysis of data collected from the ATTILA RCT investigating the impact of AT on institutionalisation for people with dementia living at home in 11 Council with Adult Social Service Responsibilities (CASSR) areas across England [30].

### Population characteristics

Three categories of population characteristics have been shown to have an impact upon healthcare utilisation namely predisposing, enabling and need categories [15]. These data included participant risk of wandering and safety which were categorised by a health or social care professional as part of the primary RCT study according to information elicited during the needs assessment. The RCT research practitioners based this categorisation on the following guidance provided by the trial manager; “in general, if there have been no or very few relevant incidents, the risk will be rated low, if they have occurred occasionally the risk will be rated moderate, and if there are frequent or very serious incidents, the risk will be high”. Level of caregiver support was categorised according to the number of times the caregiver was present; (1) live-in caregiver, (2) caregiver visits at least once / day, or (3) caregiver visits less than once/ day. Living situation was categorised as (1) living with spouse/ partner, (2) living alone or (3) Other. All participants categorised as “other” were living with another person who was not their spouse or partner, generally another relative.

RCT Practitioners, with health or social care profession backgrounds, administered the MMSE with participants at baseline. MMSE was scored on a scale which ranged from 0 to 30, where 30 indicates no dementia; scores of 26-29 indicate questionable dementia; 21-25 indicates mild dementia; 11-20 suggests moderate dementia and a score of 0-10 indicates severe dementia [31]. MMSE [29] is commonly considered during the diagnostic procedure for dementia, and meta-analysis indicates it is 85% accurate in identifying people with dementia [32]. It is a useful tool in severe conditions, but results should be considered alongside other contextual information [32]. Other tools such as Mini-cog [33] and Montreal Cognitive Assessment (MoCA) [34] appear more sensitive in detecting mild cognitive impairment (MCI) [35, 36], and may be less influenced by the level of education of the participant [37].

Assessment of need was conducted in line with normal CASSR practice to determine level of need and required AT services. AT considered in this study was installed according to routine practice, within six months of recruitment. Two practitioners on the primary RCT, with experience in dementia care and AT, collaboratively classified each item of installed AT, according to the list of installed AT categories provided in Table 1 [38]. The installation of AT reflects normal CASSR practice and was not funded, assessed or installed by the RCT [30].

**Table 1:** Categories of assistive technology

**Basic AT**	
Pendant alarm	
Non-monitored smoke detector	
Non-monitored carbon monoxide	
Key safe	
Activity monitors assessment only	
Other devices	
**Reminder or prompting devices**	
Date and time reminders	
Item locator devices	
Medication reminders/dispensers	
Voice recorders and memo minders	
Other reminder/prompting devices	
**Devices to promote safety**	
Activity monitors - on-going monitoring	
Fall detectors	
Continence management devices	
Alarm and pager units	
Flood detectors and water temperature monitor	
Gas detectors	
Monitored carbon monoxide detectors	
Monitored smoke detectors	
Monitored extreme temperature sensors	
Lighting devices	
Other safety and security devices	
**Safer walking technologies**	
To locate the user	
To alert the responder to movement	
**Communication devices**	
Intercoms	
Telephones	
Communication aids	
Other communication devices	
**Devices that support meaningful use of leisure time**	
Computer aids	
Dementia friendly TV/radio/music players	
Electronic photo albums/electronic reminiscence aids	
Electronic games	
Other devices -support meaningful use of leisure time	

### Methodology

To identify groups of people with dementia who have similar AT needs this study employed cluster analysis. This technique is essentially concerned with discovering intrinsic discrete groups within data [39–41]. Reduction of a heterogeneous sample into a number of more homogeneous groups provides a means to organise large quantities of information and facilitates consideration of multiple characteristics [42]. A Partitioning Around Medoids (PAM) algorithm for clustering data was employed due to its toleration of Gower distance to measure dissimilarity [43]. Gower distance assesses partial dissimilarities and can accommodate mixed data types [44]. The number of clusters was determined through examination of the silhouette coefficient [45]. Observations with large silhouette width (almost 1) can be considered well clustered. Silhouette width was used as a means to evaluate the clustering solution relative to other possible clustering solutions and facilitated the selection of the most robust solution.

Utility of the clustering solutions was tested through exploration of their relationship with installed AT. Installed AT data was stratified according to each of the clustering solutions in turn. The wandering cluster solution included data describing caregiver support, living situation, MMSE and level of risk of wandering of the participants. Similarly, the safety cluster solution included data describing caregiver support, living situation, MMSE and level of safety risk of the participants. Installed AT data was also stratified according to wandering risk, and safety risk for comparison purposes. Where data was available the strength of these associations between installed AT and level of wandering, safety risk and clustering solutions were tested using Chi square analyses [46].

These analyses were conducted using R Studio Software [47, 48], and the Cluster package [49].

### Ethics

Approval for this secondary data analysis study was obtained from Queen Margaret University Ethics Committee.

## Results

**Fig. 1:**
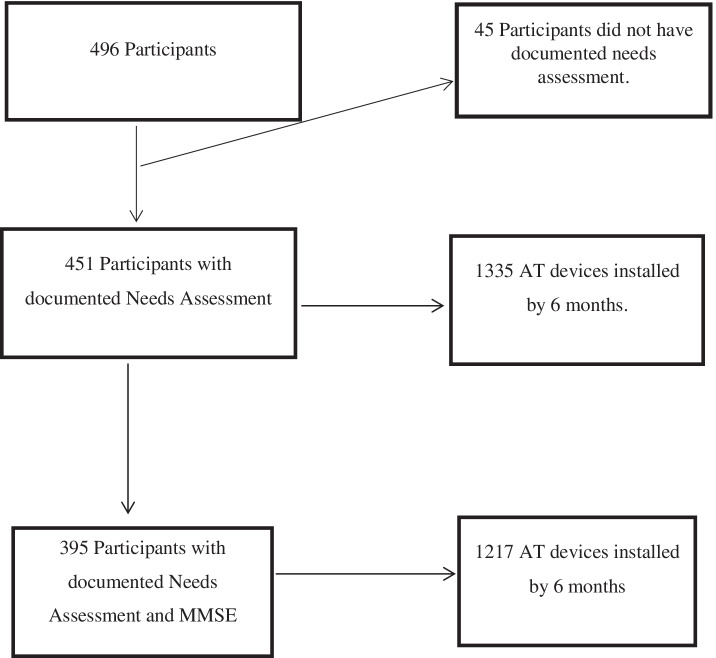
Participants with documented needs and installed AT. *MMSE* Mini Mental State Examination [29], *AT* Assistive Technology

The dataset contained anonymised information on 451 participants with dementia or suspected dementia living at home in England who had a documented needs assessment available for analysis (Fig. 1). Fifty-six participants did not have documented MMSE scores for unspecified reasons (Table 2) and were excluded from the analysis. These excluded participants were more likely to have high risk of wandering and high safety risk when compared with the remaining population. The relationship between MMSE and wandering or safety risk were not significant although participants with low risk of wandering had higher MMSE scores (M=19.05 (SD=6.1)) than participants with high risk of wandering (M=14.35 (SD=7.1)). Similarly, participants with low safety risk had higher MMSE score (M=18.35 (SD=6.66)) than participants with high safety risk (M=16.15 (SD=8.35)). This indicates that participants with reduced MMSE scores experience higher levels of wandering and safety risks.

**Table 2:** Population with and without mini mental state examination score

	Without MMSE		With MMSE	
	*n*	*%*	*n*	*%*
Gender = Female	35	62.5	229	57.97
Living situation				
Living alone	21	37.5	182	46.1
Living with spouse/partner	21	37.5	160	40.5
Other	14	25.0	53	13.4
Caregiver support				
Caregiver visits at least once per day	16	28.6	95	24.1
Caregiver visits less than once per day	11	19.6	107	27.1
Live-in caregiver	29	51.8	193	48.9
Risk of wandering				
Low	35	62.5	293	74.2
Moderate	14	25.0	76	19.2
High	7	12.5	26	6.6
Safety risk				
Low	19	33.9	211	53.4
Moderate	28	50.0	158	40.0
High	9	16.1	26	6.6

*Note*. *N =* 451, *MMSE* Mini Mental State Examination [29], *M* Mean, *SD* Standard Deviation

Overall, 1335 AT devices were installed during the 6-month period after baseline. Participants with MMSE scores (*n*=395) included within this analysis, had 1217 AT devices installed during this period (Fig. 1).

Clustering solutions including both safety and wandering risk data together with caregiver support, living situation and MMSE score had an average silhouette width below 0.5 indicating that these structures were not robust. Therefore, two separate clustering solutions were developed based on the following data variables: Risk of Wandering or Safety Risk, Caregiver Support, Living Situation and MMSE score. These will now be described in turn.

Table 3 provides a summary of the characteristics of the participants included in each of the three clusters based upon caregiver support, MMSE, living situation and risk of wandering data. Clusters were named to reflect dominant characteristics of participants within that cluster [41]: (1) “Living with Spouse/ Partner”, (2) “Living with Other” and (3) “Living alone”.

**Table 3:** Partitioning around medoids (PAM) summary for wandering cluster

Cluster	Caregiver support			MMSE		Living situation			Risk of wandering		
		n	%				n	%		n	%
1. Living with Spouse/ Partner (*n* = 156)	Live-in caregiver: Caregiver visits at least once per day: Caregiver visits less than once per day:	147 8 1	94.2 5.1 0.6	Min: 1^st^ Qu.: Median: *M*: 3^rd^ Qu.: Max:	0.0 13.0 19.0 17.6 24.0 28.0	Living alone: Living with Spouse/ Partner: Other:	0 156 0	0 100 0	Low: Moderate: High:	112 34 10	71.8 21.8 6.4
2. Living with Other (*n* = 51)	Live-in caregiver: Caregiver visits at least once per day: Caregiver visits less than once per day:	46 4 1	90.2 7.84 1.78	Min: 1^st^ Qu.: Median: *M*: 3^rd^ Qu.: Max:	0.0 14.5 18.0 17.6 24.0 29.0	Living alone: Living with Spouse/ Partner: Other:	0 0 51	0 0 100	Low: Moderate: High:	36 10 5	70.6 19.6 9.8
3. Living Alone (*n* = 188)	Live-in caregiver: Caregiver visits at least once per day: Caregiver visits less than once per day:	0 83 105	0 44.2 55.8	Min 1^st^ Qu. Median *M* 3^rd^ Qu. Max	0.0 15.0 20.0 18.8 23.0 28.0	Living alone: Living with Spouse/ Partner: Other:	182 4 2	96.8 2.1 1.1	Low: Moderate: High:	145 32 11	77.1 17.0 9.0
Medoids for Wandering Clusters											
1. Living with Spouse/ Partner	Live-in caregiver				19	Living with spouse/partner			Low		
2. Living with Other	Live-in caregiver				18	Other			Low		
3. Living Alone	Caregiver visits less than once per day				20	Living alone			Low		

*Note*. *N* = 395, *PAM* Partitioning Around Medoids, *MMSE* Mini Mental State Examination [29], *Min* Minimum, *Qu* Quarter, *M* Mean, *Max* Maximum

**Fig. 2:**
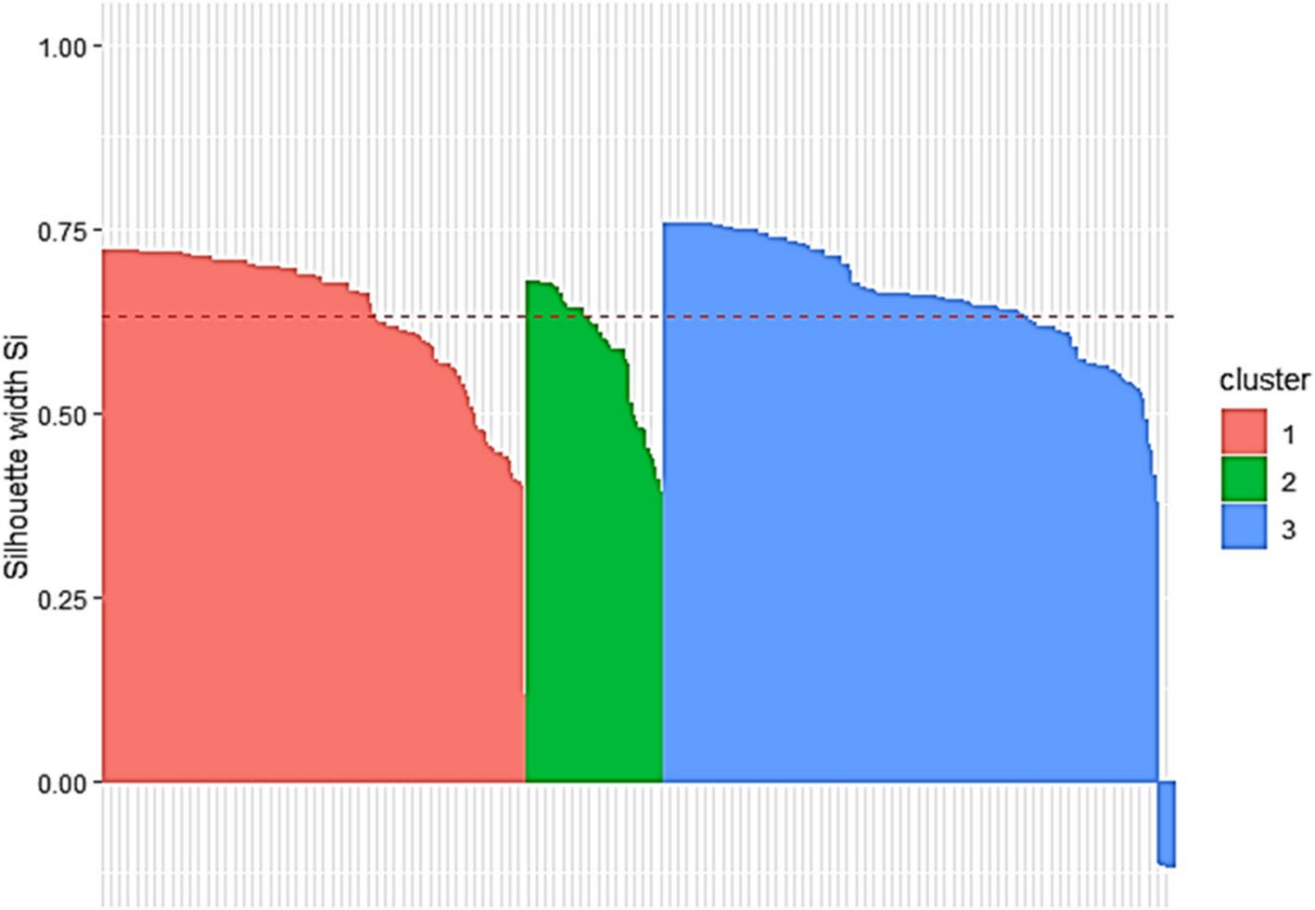
Silhouette Plot for Wandering Cluster (Average Silhouette Width: 0.63)

Six participants included in this third cluster exhibited negative silhouette width [45] (Fig. 2). These participants were unusual within this dataset as they lived with spouse or partner or other yet did not have a live-in caregiver. Due to their small number it was not possible to draw conclusions regarding the Assistive Technology installations for these participants. This clustering solution had an average silhouette width of 0.63 indicating that a reasonable structure has been found [50]. Medoids or exemplars are also presented for each cluster.

Table 4 provides a summary of the characteristics of the participants based upon caregiver support, MMSE, living situation and safety risk data. Clusters were named (1) “Live with Someone”, and (2) “Live-out Caregiver” to reflect characteristics of their participants [41].

**Table 4:** Partitioning around medoids (PAM) summary for safety clusters

Cluster	Caregiver support			MMSE		Living situation			Safety risk		
		n	%				n	%		n	%
1. Live with someone (*n* = 208)	Live-in Caregiver: Caregiver visits at least once per day: Caregiver visits less than once per day:	193 12 3	92.79 5.77 1.44	Min.: 1^st^Qu.: Median: *M*: 3^rd^ Qu.: Max.:	0.00 14.00 18.00 17.66 24.00 29.00	Living alone: Living with spouse/ partner: Other:	0 158 50	0.0 75.96 24.04	Low: Moderate: High:	121 70 17	58.17 33.65 8.17
2. Live out caregiver (*n* = 187)	Live-in Caregiver: Caregiver visits at least once per day: Caregiver visits less than once per day:	0 83 104	0.0 44.38 55.61	Min.: 1^st^ Qu.: Median: *M:* 3^rd^ Qu.: Max.:	0.00 15.00 20.00 18.72 23.00 28.00	Living alone: Living with spouse/ partner: Other:	182 2 3	97.33 1.07 1.60	Low: Moderate: High:	90 88 9	48.13 47.06 4.81
Medoids for safety clusters											
1. Live with someone	Live-in Caregiver			18		Living with spouse/ partner			Low		
2. Live out caregiver	Caregiver visits less than once per day			20		Living alone			Moderate		

*Note*. *N* = 395, *PAM* Partitioning Around Medoids, *MMSE* Mini Mental State Examination [29], *Min* Minimum, *Qu* Quarter, *M* Mean, *Max* Maximum

**Fig. 3:**
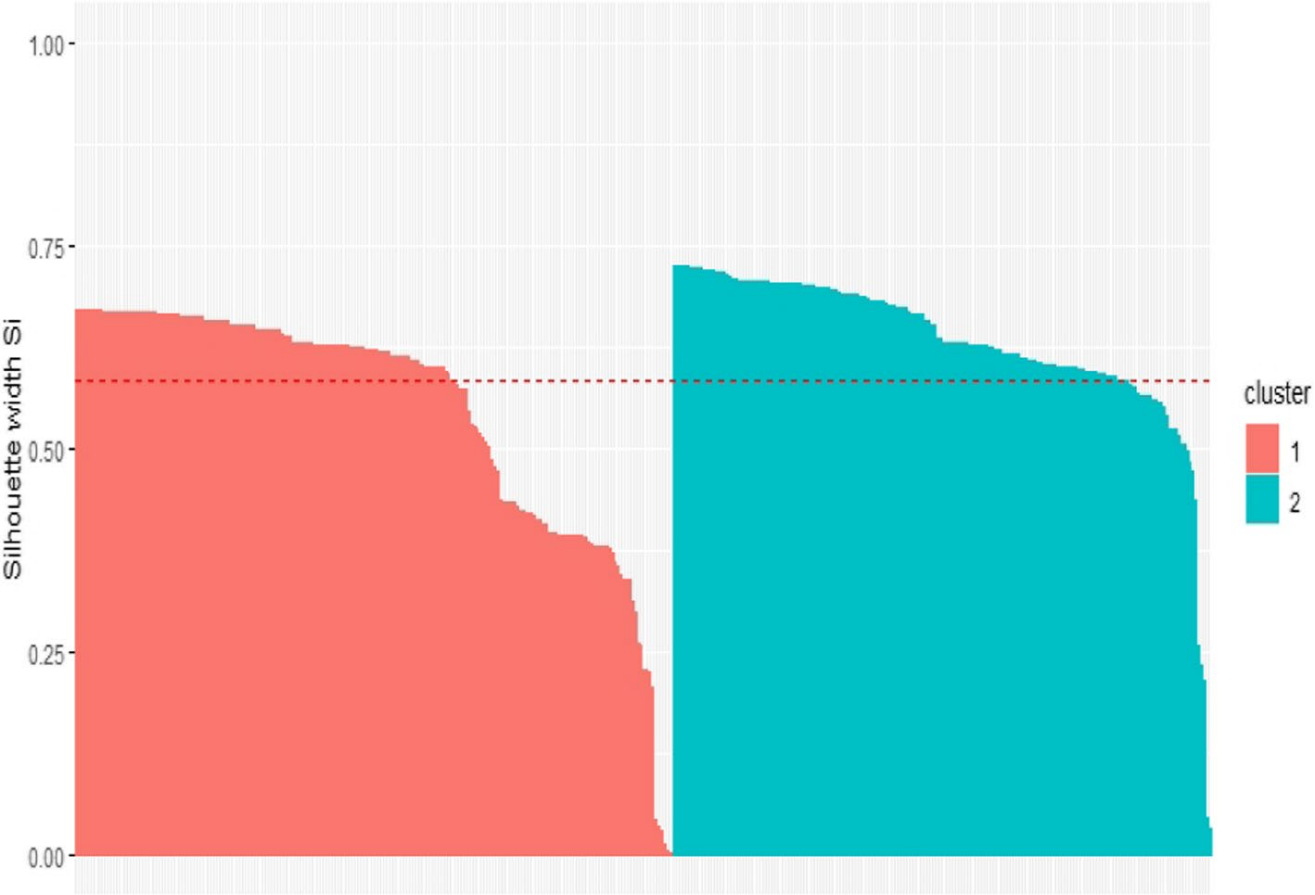
Silhouette Plot for Safety Cluster (Average Silhouette Width: 0.59)

All participants had positive silhouette widths in this solution (Fig. 3). This average silhouette width of 0.59 indicates that a reasonable structure has been identified [50]. Again, medoids are presented for each cluster.

Associations between the clusters and categories of installed AT are presented in Table 5, together with associations identified between installed AT and risk of wandering or safety risk.

**Table 5:** Associations with installed assistive technology

Risk	Low			Moderate			High		
		*χ*^*2*^	*p*		*χ*^*2*^	*p*		*χ*^*2*^	*p*
**Wandering (*N*** **= 451)**	Medication Reminders and Dispensers Pendant Alarms	13.18 7.79	.001** .02**	Activity Monitors for Ongoing Monitoring Safer Walking Technologies to Locate the User	15.78 39.04	<.001*** <.001***	Intercoms Safer Walking Technologies to alert a Responder of Movement Telephones	27.90 40.40 13.51	<.001*** <.001*** .001**
**Safety (*N*=451)**	Safer Walking Technologies to Locate the User	13.41	.001**	NA			Fall Detectors	68.62	<.001***
**Wandering cluster (*N*=395)**	**Living with Spouse/ partner**			**Living with Other**			**Living Alone**		
	Fall Detectors Safer Walking Technologies to alert a responder of movement	6.94 7.33	.03* .02*	Medication Reminders and Dispensers	15.91	<.001***			
**Safety cluster (*N*=395)**	**Live with Someone**			**Live Out Caregiver**					
	Safer Walking Technologies to alert a Responder of Movement Safer Walking Technologies to Locate the User	19.67 21.96	<.001*** <.001***	Monitored Smoke Detectors Pendant Alarms	7.58 10.42	.006** .001**			

*Note*. *AT* Assistive Technology, *NA* Not available, * *p* < .05, ** *p* < .01, *** *p* < .001, associations are presented under group which most frequently received this category of AT

Risk of wandering was associated with installation of safer walking technologies to alert a responder of movement *χ*^2^ (2, *N*=451) =40.40, *p*<.001), safer walking technologies to locate the user *χ*^2^ (2, *N*=451) = 39.04, *p*<.001, medication reminders and dispensers *χ*^2^ (2, *N*=451) =13.18, *p*=.001, telephones *χ*^2^ (2, *N*=451) =13.51, *p*=.001, intercoms *χ*^2^ (2, *N*=451) =27.90, *p*<.001, pendant alarms *χ*^2^ (2, *N*=451) =7.79, *p*=.02 and activity monitors for ongoing monitoring χ^2^ (2, *N*=451) =15.78, *p*<.001) (Table 5). Pendant alarms and medication reminders and dispensers were most frequently installed for participants with low risk of wandering. Activity Monitors for Ongoing Monitoring and Safer walking technologies to locate the user were most frequently installed for participants with moderate risk of wandering. Intercoms, Telephones and Safer Walking Technologies to alert a Responder of Movement were most frequently installed for participants with high risk of wandering.

Safety risk was associated with installation of safer walking technologies to locate the user *χ*^2^ (2, *N*=451) =13.41, *p*=.001 most frequently installed for people with low safety risk; and fall detectors *χ*^*2*^ (2, *N*= 451) =68.62, *p*<.001 which were most frequently installed for people with high safety risk.

The wandering cluster solution was associated with installation of fall detectors *χ*^2^ (1, *N*=395) =6.94, *p*=.03, safer walking technologies to alert a responder of movement *χ*^2^ (2, *N*=395) =7.33, *p*=.02 and medication reminders and dispensers *χ*^2^ (2, *N*=395) =15.91, *p*<.001.

The safety cluster solution was associated with safer walking technologies to alert a responder of movement χ^2^ (1, *N*=395) =19.67, *p*<.001 and safer walking technologies to locate the user *χ*^2^ (1, *N*=395) =21.96, *p*<.001. Both types of safer walking technologies were most frequently installed for participants “living with someone”. Monitored smoke detectors χ^2^ (1, *N*=395) =7.58, *p*=.006 and pendant alarms χ^2^ (1, *N*=395) = 10.42, *p*=.001) were also associated with the safety cluster solution and were most frequently installed for participants in the “live out caregiver” cluster.

## Discussion

This study has developed understanding of the interaction of heterogeneous person characteristics including predisposing characteristics, needs and enabling resources, and their impact upon installed AT interventions in current practice. Results demonstrate that robust clusters created from data describing the characteristics of people with dementia can provide a basis for the exploration of the impact of multiple factors upon AT installations for this population. Subsequently this study validated these cluster solutions through demonstration of their applicability to data describing AT installed for people with dementia living at home.

Cluster analyses appear to have grouped people with dementia according to their caregiver support and living situation, although MMSE and risk of wandering or safety risk were also considered. Subsequent analysis of the relationship between the cluster solutions and installed AT illustrated differences in patterns of AT installation in regard to safety and wandering risk indicating that these are associated with contrasting areas of concern. AT provided to mitigate safety risk suggests consideration of mobility issues, including falls. Whereas, installed AT associated with level of wandering risk is more varied perhaps because of a wider area of interest. Associations between installed AT and clustering solutions in this study indicate that the relationship between the person with dementia and their caregiver or support network may also influence AT provision in a number of ways. These include that; (1) AT is provided to meet the needs of the caregiver; (2) input from the caregiver is required to obtain, maintain or monitor AT; and/ or (3) the caregiver provides a different view of the needs of the person with dementia resulting in a change in AT provision. These will now be discussed in turn.

Installation of safer walking technologies to alert a responder of movement were associated with the “living with spouse/ partner”, or the “live with someone” clusters. Additionally, installation of safer walking technologies to locate the user was associated with the “live with someone” cluster. This type of AT may be used by caregivers to track people with dementia who are perceived to have lower risk of becoming lost and as a back up to caregiver support [51]. However, as GPS technologies are generally used to back-up other forms of support and rarely facilitate independent walking for the person with dementia [51], results indicate that AT provision may be influenced by the needs of caregivers, such as fear of losing the person with dementia, to improve quality of life, and reduce stress [52]. This confirms previous studies indicating a reduction in caregiver anxiety following the installation of AT [52, 53]. Safety is a known concern for caregivers even when the person with dementia is unable to leave the home [51], and often leads to restrictions being placed upon the independent activity of the participant. Caregivers prioritise the safety of the person with dementia even above their autonomy or privacy [54]. If safer walking technologies are primarily installed to alleviate caregiver anxiety, this explains why this type of AT was less likely to be provided for people with dementia living alone. Caregiver anxiety is associated with the institutionalisation of the person with dementia; hence caregiver stress reduction has direct benefit for them and may be the reason for their acceptance of AT which restricts their autonomy [14]. This may not be the case for people with dementia living alone. AT providers are therefore required to balance the needs and rights of people with dementia, whilst also considering the needs of the caregiver [55].

Participants living with others were more likely to receive installations of fall detectors, safer walking technologies to alert a responder of movement and safer walking technologies to locate the user. Whereas, participants living alone received more basic AT items such as monitored smoke detectors, and carbon monoxide detectors. Reasons for these differences are unclear but in addition to the absence of caregivers’ concerns, may include there being no-one to adapt, monitor or respond to AT on their behalf [56]. Caregivers who live with the person with dementia are likely to be able to respond more quickly to alerts than monitoring centres. As, wandering incidents may occur frequently, they can require high levels of response which are unavailable from formal response teams. Familiar caregivers will also have more understanding of the particular requirements of the person with dementia [57].

Further, caregivers and co-residents may influence the assessment process, and therefore the AT installed for people living with others. Decisions to use tracking technologies have been shown to be informed by the caregivers’ personal assessment of the safety of the participant [51]. Additionally, caregivers report higher levels of need than people with dementia report themselves [27]. Results indicate a focus on the priorities of caregivers rather than people with dementia. People with dementia identify daily activities and socialising as their priority [58]. Focussing on activities which increase participation can increase wellbeing, and reduce anxiety related behaviour such as wandering [28].

In such incidences, people with dementia living with others are more likely to have a caregiver who is able to provide an overview of their needs and abilities on their behalf [59, 60].

Results of this study indicate that there are factors other than safety or wandering risk, which can affect installation of AT. Factors which may not be considered during the AT needs assessment include the impact of caregiver needs, the caregiver’s view of the person with dementia’s needs or the support received from informal caregivers. This may reflect limitations in the skills and knowledge of staff conducting the assessment of need [3, 60]. People living alone are less likely to be diagnosed with dementia, and clinicians often struggle to identify their needs [59, 60]. Additionally, people with more severe impairment often have less documented assessment than people with milder cognitive impairment [10]. People with moderate to severe dementia may have difficulty understanding questions in assessment tools [61]. Poor vision and hearing, deficient schooling and consequences of stroke or tremor may also make the completion of assessments difficult [62]. Overall, this suggests that needs which are perhaps considered difficult to assess or cannot be directly observed such as psychological needs often remain unassessed [10]. People with dementia living with others may be more likely to have a caregiver who can provide an overview of their needs and abilities. Whereas, the reduced level of understanding of the needs of particular groups such as people with dementia living alone, results in them being less likely to receive services, despite being identified as a high-risk group [63]. In order to account for the AT needs of people at all stages of dementia there is a requirement for the development of skilled assessors, validated assessment tools and alternative methods of assessment. Further, these groups are also less likely to be included in research which would advance understanding of their intervention requirements [59].

As caregivers have been identified as key actors in ensuring the safety of people with dementia [64], it is important to consider their views during the assessment of the person with dementia. Caregivers often monitor and maintain AT on behalf of the person with dementia. Additionally, AT is often provided for the reassurance and support of caregivers [65]. It is therefore, difficult to distinguish between the needs of people with dementia and their caregivers as these are interwoven in a complex manner due to multiple interdependencies between these groups [16]. Consideration of the views, needs and capabilities of the person with dementia and their caregiver or people they live with, during the assessment process will provide a target for the tailoring of interventions and increase the ability to meet needs.

This study indicates limitations in the needs assessment of people with dementia, particularly those with moderate or severe impairment. There is a requirement to develop validated assessment tools which consider the needs of people with more severe dementia, or who also experience communication difficulties [60]. Additional training in the assessment of people with dementia should be available to clinicians working in this field to facilitate the development of expertise. Results also indicate assessors have limited understanding of the relationship between personal characteristics and AT [2], which may be due to organisational policy, or limitations of time, support, training, knowledge and resources [66]. Additionally, a supply led allocation process or preoccupation with risk generated interventions restricts choice and may increase distress [10, 67]. Policies, staff training and resources should be reviewed to ensure that they support person centred care. Assessment should focus on the individuality of the person with dementia and their circumstances, thereby increasing the acceptability of person centred rather than supply led AT interventions [10]. Stakeholders will need to ensure access to sufficient AT resources to enable the installation of appropriate AT to meet identified needs [10].

### Limitations

Limitations of this study include the low number of participants with moderate to severe dementia, high safety risk or high wandering risk. This restricts the transferability or generalisability of results, and further research is required to validate results for these populations [68]. Safety risk and wandering risk and AT were categorised according to non-validated criteria. The reliability and validity of these instruments is therefore uncertain and restricts comparisons of these results with further research [69]. MMSE does not always accurately discriminate between the stages of dementia [25]. Limited sample numbers also meant it was not possible to further validate the cluster analysis solutions on additional data [70].

## Summary

This study has explored the impact of multiple factors upon AT installed for people with dementia living at home and provides validation of the use of partitioning around medoids cluster analysis as a method within this field. Results indicate that installation of AT for people with dementia living at home is influenced not only by their level of safety or wandering risk, but also by the level of caregiver support they receive and their living situation. There are a number of changes required to facilitate dementia friendly person-centred care. Policies should support assessment which considers the needs of the person with dementia, their caregivers and other members of their social network before installing AT. In order to improve effectiveness of AT interventions for people with dementia living at home there is a requirement for educators and professional bodies to advance assessment practice through mentorship and training. Assessors require to develop validated comprehensive assessment tools which account for different circumstances and impairments often experienced within this population, and which consider a wide range of care needs including psychological and social needs. There is also a requirement for assessment tools to direct assessors towards appropriate interventions [71], through evaluation of a wide range of needs experienced both by the person with dementia and members of their support network. There is a requirement for a wider range of AT to be available for installation in order to meet the individual needs of people with dementia and their caregivers.

## Data Availability

The dataset analysed during this study is available from robert.howard@ucl.ac.uk on request.

## Abbreviations

AT: Assistive Technology/ Assistive Technologies
CASSR: Council with Adult Social Service Responsibilities
GPS: Global Positioning System
MMSE: Mini Mental State Examination
PAM: Partitioning Around Medoids
RCT: Randomised Controlled Trial
