# Correction to: Differences in assistive technology installed for people with dementia living at home who have wandering and safety risks

**DOI:** 10.1186/s12877-021-02616-w

**Published:** 2022-03-01

**Authors:** Eleanor Curnow, Robert Rush, Sylwia Gorska, Kirsty Forsyth

**Affiliations:** School of Health Sciences, Queen Margaret University, Edinburgh, EH21 6UU UK

**Correction to: BMC Geriatr 21, 613 (2021)**

**https://doi.org/10.1186/s12877-021-02546-7**

After publication of this article [1], the authors reported that the wrong figure appeared as Fig. 1; the figure should have appeared as shown below.

**Fig. 1:**
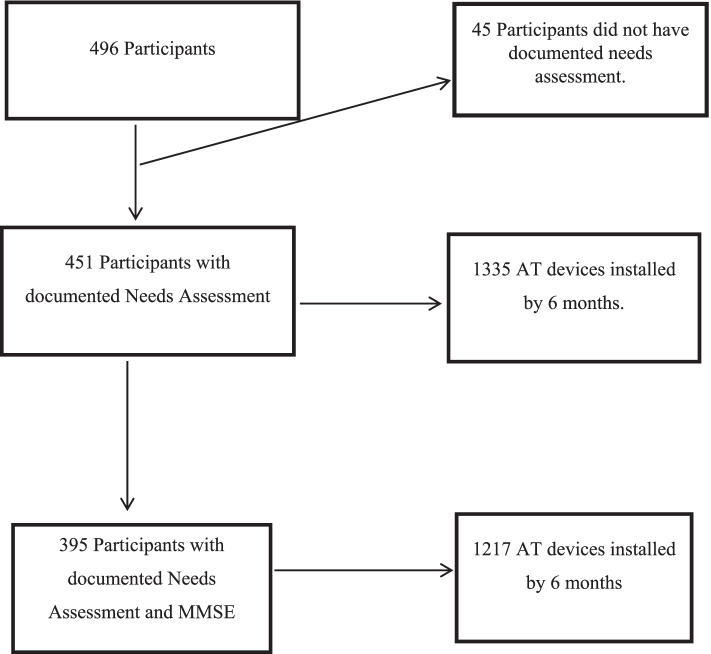
Participants with documented needs and installed AT. MMSE = Mini Mental State Examination [29]; AT = Assistive Technology

The original article [1] has been updated.
